# Post hemorrhagic hydrocephalus and neurodevelopmental outcomes in a context of neonatal intraventricular hemorrhage: an institutional experience in 122 preterm children

**DOI:** 10.1186/s12887-018-1249-x

**Published:** 2018-08-31

**Authors:** Vianney Gilard, Alexandra Chadie, François-Xavier Ferracci, Marie Brasseur-Daudruy, François Proust, Stéphane Marret, Sophie Curey

**Affiliations:** 1 Neurosurgery Department, Rouen University Hospital, 1 rue de Germont, 76000 Rouen, France; 2 Paediatrics Department, Rouen University Hospital, 76000 Rouen, France; 3 Department of Radiology, Rouen University Hospital, 76000 Rouen, France; 4 Neurosurgery Department, Strasbourg University Hospital, 67000 Strasbourg, France

**Keywords:** Intraventricular hemorrhage, Neonatal, Hydrocephalus, Neurodevelopmental outcomes

## Abstract

**Background:**

Intraventricular hemorrhage (IVH) is a frequent complication in extreme and very preterm births. Despite a high risk of death and impaired neurodevelopment, the precise prognosis of infants with IVH remains unclear. The objective of this study was to evaluate the rate and predictive factors of evolution to post hemorrhagic hydrocephalus (PHH) requiring a shunt, in newborns with IVH and to report their neurodevelopmental outcomes at 2 years of age.

**Methods:**

Among all preterm newborns admitted to the department of neonatalogy at Rouen University Hospital, France between January 2000 and December 2013, 122 had an IVH and were included in the study. Newborns with grade 1 IVH according to the Papile classification were excluded.

**Results:**

At 2-year, 18% (*n* = 22) of our IVH cohort required permanent cerebro spinal fluid (CSF) derivation. High IVH grade, low gestational age at birth and increased head circumference were risk factors for PHH. The rate of death of IVH was 36.9% (*n* = 45). The rate of cerebral palsy was 55.9% (*n* = 43) in the 77 surviving patients (49.4%). Risk factors for impaired neurodevelopment were high grade IVH and increased head circumference.

**Conclusion:**

High IVH grade was strongly correlated with death and neurodevelopmental outcome. The impact of an increased head circumference highlights the need for early management. CSF biomarkers and new medical treatments such as antenatal magnesium sulfate have emerged and could predict and improve the prognosis of these newborns with PHH.

**Electronic supplementary material:**

The online version of this article (10.1186/s12887-018-1249-x) contains supplementary material, which is available to authorized users.

## Background

Intraventricular hemorrhage (IVH) remains a serious complication in premature children, affecting approximately 20–30% of infants born < 29 weeks estimated gestational age (EGA) [1–3]. In a few cases, IVH can occur in fetus during pregnancy or in children born at term. Improvements in obstetric care have led to an increase in survival and a decrease in the incidence of IVH in preterm newborns [4] secondary to the antenatal administration of corticosteroid and/or sulfate magnesium. Nevertheless, a correlation has been established between low gestational age at birth and the incidence and severity of IVH [5].

In preterm newborns, the physiopathology [6–8] of bleeding is based on hemorrhagic transformation of hypoxia-ischemia in the vulnerable subependymal germinal matrix. This location is fed by rich terminal vascularization with an intense metabolism, immature at this step of brain development and highly sensitive to hemodynamic fluctuations. The invasion of bleeding in the ventricular system is responsible for post-hemorrhagic hydrocephalus (PHH) [9] due to the obstruction of cerebrospinal fluid (CSF) circulation and to the inflammatory response of the ependyma causing a loss of compliance and finally a decrease of CSF reabsorption. Moreover, white matter lesions due to intraparenchymal hemorrhage are responsible for alteration of oligodendrocytes and astrocytes, affecting the myelination and organization of the cerebral cortex.

Despite many treatment options, there is still no consensus on the management of PHH and very few data about neurodevelopmental outcomes and predictive factors of PHH [3, 10, 11] . The indication and the timing of surgical treatment [12, 13] remain challenging for the neurosurgeon and the neonatologist, as does the impact of IVH on the neurodevelopmental evolution of the child. The objective of this study was to evaluate the predictive factors of evolution to PHH in 122 newborns with neonatal IVH, and report their neurodevelopmental outcomes at 2 years.

## Methods

### Baseline demographic data

All preterm newborns who were admitted to the neonatal intensive care unit of the level III maternity wing at Rouen University Hospital between January 2000 and December 2013 and who had a neonatal IVH were included in the study. Infants with major malformations or syndromes, including central nervous system defects, congenital cardiopathies, gastrointestinal defects, and chromosomal abnormalities, were excluded. Maternal and neonatal information from birth to death or hospital discharge were collected in the medical charts and included gender, gestational age, birth weight, head circumference (HC), administration of antenatal magnesium sulfate and steroids, placement of a shunt for PHH and the type of device used, timing of surgery, the occurrence of meningitis and IVH grade.

IVH was defined on the basis of Papile’s criteria [14] on cranial ultrasound (cUS) performed in all preterm newborns during the first week of life in the absence of clinical signs according to the following criteria: Grade 1: hemorrhage confined to the germinal matrix, Grade 2: extension of hemorrhage into lateral ventricles without ventricular dilatation, Grade 3: ventricular hemorrhage with ventricular dilatation, Grade 4: parenchymal hemorrhage. Patients with isolated grade 1 IVH were excluded from the study because it is a frequent situation in preterm child before 30 weeks of gestation (WG) and grade 1 IVH are not associated with PHH witout intraventricular bleeding.

The primary outcome was the rate of PHH in preterm newborns with neonatal IVH. Secondary criteria were neurodevelopmental outcomes at 2 years of corrected age considering motor impairment such as cerebral palsy or sensorial disorders, risk factors for impaired clinical evolution at 2 years and predictive factors of evolution to PHH.

### Outcome definitions

#### Primary outcome

PHH was defined as clinical signs of increased intracranial pressure, including increased HC > + 2 Standard Deviation (SD), bulging anterior fontanel, splayed cranial sutures, strabismus, decline in neurological examination, poor feeding, lethargy, and irritability accompanied by progressive ventricular dilation noted on serial cUS requiring CSF shunt.

#### Secondary outcomes

Mortality rate was assessed during the two years of follow-up.

Gross motor function was assessed at 24 months of corrected age by the five level Palisano’s Gross Motor Function Classification System (GMFCS) [15] performed by trained neuropediatricians at Rouen University Hospital. GMFCS ≥2 indicated adverse motor evolution.

Language development was assessed by the association of words at 24 months of corrected age using the MacArthur questionnaire [16]. Adverse language development was defined as the absence of words association at the age of 24 months.

Severe visual impairment was defined as bilateral acuity < 0.3. Deafness was defined as bilateral permanent hearing loss requiring amplification.

### Statistical analyses

Unadjusted comparisons of neonatal characteristics, IVH grading and patients care between positive and impaired neurodevelopmental outcomes were made using chi-square or Fisher’s exact tests for categorical data and two-sided t-tests for continuous data. Significant univariate variables were included in the multivariate logistic regression model and excluded in a forward stepwise fashion by least-significant variable until all included variables had *p* < 0.05.

#### Ethical approval

All procedures performed in studies involving human participants were in accordance with the ethical standards of the institutional and/or national research committee and with the 1964 Helsinki Declaration and its later amendments or comparable ethical standards.

## Results

### Demographic data

During the 14 years of the study, 122 newborns (sex ratio M/F 1.1) met the inclusion criteria (Additional file 1) and had one IVH at least. Median gestational age at birth (WG) was 28 WG (min: 23-max: 35). Demographic data are presented in Table 1. Concerning clinical presentation, 28 newborns (22.9%) were asymptomatic, 43 (35.2%) presented with hypotonia, 11 (9%) had a bulging fontanel, 86 (70.5%) had an increased head circumference > + 2 SD and 16 (13.1%) presented with epilepsy. At radiological examination based on ultrasound and Papile’s criteria, 52 newborns (42.6%) had grade 2 IVH, 22 (18%) had grade 3 IVH and 48 (39.3%) had grade 4 IVH.

**Table 1:** Demographic data

Number of patients	122 (%)
Sexe	
Male	64
Female	58
Sex ratio (M/F)	1.1
Term	
Premature	122 (100)
Mean gestational age (weeks)	29.6 +/− 4.8
Etiology of prematurity	
induced (antenatal diagnosis)	7 (5.7)
maternal hypertension	11 (9)
preterm premature rupture of the membranes	56 (45.9)
placenta previa, other hemorrhage	10 (8.2)
infection	12 (9.8)
undetermined	26(21.3)
Antenatal administration	
corticosteroids (single dose)	34 (27.9)
corticosteroids (2 doses)	30 (24.6)
magnesium	16 (13.1)

### Primary outcome

During the study period, 22 newborns (18%) developed symptomatic PHH. Among these 22 newborns, 6 had initially presented a grade 4 hemorrhage, 10 a grade 3 hemorrhage and 6 a grade 2 hemorrhage, according to the Papile classification. In these 22 newborns, ventriculoperitoneal shunt (VPS) was the first device to be implanted in 7 cases; secondary to other devices in 15 cases. When another device was implanted first, it consisted in ventriculo subgaleal shunts (VSGS) in 10 cases, external ventricular drainage (EVD) in 3 cases or ventriculocysternostomy in 2 cases. On multivariate analysis, risk factors for long-term PHH were high IVH grade on cUS and an increased HC > + 2 SD at diagnosis.

Other variables with their respective odds ratio are presented in Table 2 (univariate analysis) and Table 3 (multivariate analysis).

**Table 2:** Risk factors for post hemorrhagic hydrocephalus on univariate analysis

		Total		PHH		No PHH		*P* value	test
Variables	Modalities	n	%	n	%	n	%		
Papile grading	2	52	42,62	6	27,27	46	37,70	0,0011	chi2
	3	22	18,03	10	45,45	12	9,84		
	4	48	39,34	6	27,27	42	34,43		
increased head circumference > +2SD	Yes	86	70,49	20	16,39	66	54,10	0,0204	chi2
	No	36	29,51	2	1,64	34	27,87		
Gestational age at birth (WA)	< 30	67	54,91	14	11,47	61	50,0	0,031	Fisher
	30–37	55	45,08	8	6,56	39	31,97		
Birth weight (percentiles)	0–24	53	43,44	11	9,02	42	34,43	0,0047	Fisher
	25–49	9	7,38	2	1,64	7	5,74		
	50–74	27	22,13	6	27,27	21	17,21		
	75–100	33	27,05	3	13,64	30	24,59		
Sex	Female	58	47,54	8	6,56	50	49,98	0,246	Chi2
	Male	64	52,46	14	11,47	50	40,98		
Magnesium administration	No	106	86,89	15	12,29	91	74,59	0,45	fisher
	Yes	16	13,11	7	5,74	9	7,38		
Corticosteroids administration	No	58	47,54	10	8,19	42	34,43	0.23	chi2
	Yes	64	52,46	12	9,84	14	11,47		

*PHH*, Post hemorrhagic hydrocephalus; *SD*, Standard deviation; *WA*, Weeks of amenorrhea

**Table 3:** Risk factors for post hemorrhagic hydrocephalus on multivariate analysis

Variables		OR	CI	p
Ultrasound grade	3 versus 2	4.06	0.99–16.63	0.001
	4 versus 2	7.22	2.08–25.08	0.003
Increased head circumference		10.2	2.17–48	0.020
Gestation	< 30WA versus 30-37WA	0.14	0.03–0.64	0.001
	30-37WA versus <37WA	0.26	0.06–1.15	0.003
Birth weight	4th quartile versus 2nd quartile	3.49	0.85–4.39	0.004
	3rd quartile versus 2nd quartile	3.33	0.23–4.09	0.007

*OR*, odds ratio; *CI*, confidence interval; *PHH*, Post hemorrhagic hydrocephalus; *SD*, Standard deviation; *WA*, Weeks of amenorrhea

### Secondary outcomes

Death occurred in 45 of the 122 infants in our cohort (36.9%). Among these 45 infants, death was due to initial bleeding in 20 (44.4%), pulmonary insufficiency in 7 (15.6%) and multiple organ failure in 18 (40%). In our cohort, risk factors for death were high IVH grade on cUS and low gestational age at birth. These variables with their respective odds ratio are presented in Table 4 (univariate analysis) and Table 5 (multivariate analysis).

**Table 4:** Risk factors of death in univariate analysis

		Total		Death		Alive at follow-up		*P* value	test
Variables	Modalities	n	%	n	%	n	%		
Papile grading	2	52	42,62	7	5,74	42	34,43	0,0001	chi2
	3	22	18,03	3	2,46	19	15,57		
	4	48	39,34	38	31,15	13	10,66		
increased head circumference > +2SD	Yes	86	70,49	40	32,79	46	37,70	0,0007	chi2
	No	36	29,51	5	4,10	31	25,41		
Gestational age at birth (WA)	< 30	80	65,57	48	39,34	35	28,69	0.0041	fisher
	30–37	42	34,43	6	4,92	33	27,05		
Birth weight (percentiles)	0–24	53	43,44	21	17,21	32	26,23		
	25–49	9	7,38	6	4,92	3	2,46	0.002	fisher
	50–74	27	22,13	8	6,56	19	15,57		
	75–100	33	27,05	10	8,20	23	18,85		
Sex	Female	58	47,54	24	19,67	39	31,97	0,33	chi2
	Male	64	52,46	21	17,21	38	31,15		
Magnesium administration	No	106	86,9	3	2,46	8	6,56	0,7447	chi2
	Yes	16	13.1	42	34,43	69	56,56		
Corticosteroids administration	No	58	47,64	10	8,20	48	39,34	0.23	chi2
	Yes	64	52,46	11	9,02	53	43,44		

*SD*, Standard deviation; *WA*, Weeks of amenorrhea

**Table 5:** Risk factors of death in multivariate analysis

Variables		OR	CI	p
Ultrasound grade	3–4 versus 2	17.31	6.25–7.98	0.001
Birth weight	4th quartile versus 2nd quartile	1.51	0.5–3.8	0.19
	3rd quartile versus 2nd quartile	4.6	0.9–22.1	0.33
Gestation	< 30WA versus 30-37WA	5.85	1.2–2.5	0.03
	30-37WA versus <37WA	3.1	0.4–2.5	0.41
Meningitis		0.61	0.1–2.4	0.67

*OR*, odds ratio; *CI*, confidence interval; *SD*, Standard deviation; *WA*, Weeks of amenorrhea

Concerning motor function of the 77 survivors at 2 years, GMFCS score was 1 in 34 (44.2%), 2 in 27 (35.1%), 3 in 10 (13%), 4 in 3 (3.9%) and 5 in 3 (3.9%) infants. 43 patients had a GMFCS ≥2 and 16 (20.8%) were non-ambulatory. Risk factors for negative evolution were high IVH grade on ultrasound and increased HC at diagnosis (Tables 6 and 7).

**Table 6:** Risk factors for pejorative motor outcomes on univariate analysis

		Total		Pejorative outcome		Favorable outcome		*P* value	test
Variables	Modalities	n	%	n	%	n	%		
Papile grading	2	52	42,62	6	27,27	46	37,7	0,0011	chi2
	3	22	18,03	10	45,45	12	9,84		
	4	48	39,34	6	27,27	42	34,43		
increased head circumference > +2SD	Yes	86	70,49	20	16,39	66	54,1	0,0204	chi2
	No	36	29,51	2	1,64	34	27,87		
Gestational age at birth (WA)	<30	67	54,91	14	11,47	61	50	0,031	Fisher
	30–37	55	45,08	8	6,56	39	31,97		
Birth weight (percentiles)	0–24	53	43,44	11	9,02	42	34,43	0,0047	Fisher
	25–49	9	7,38	2	1,64	7	5,74		
	50–74	27	22,13	6	4,92	21	17,21		
	75–100	33	27,05	3	2,46	30	24,59		
Sex	Female	58	47,54	8	6,56	50	49,98	0,246	Chi2
	Male	64	52,46	14	11,47	50	40,98		
Magnesium administration	No	106	86,89	15	12,29	91	74,59	0,45	Fisher
	Yes	16	13,11	7	5,74	9	7,38		
Corticosteroids administration	No	58	47,54	10	8,19	42	34,43	0.23	chi2
	Yes	64	52,46	12	9,84	14	11,47		
EVD	No	114	93,44	16	13,11	98	80,33	< 0,0001	Fisher
	Yes	8	6,56	6	4,92	2	1,64		
VP shunt	No	115	94,26	14	11,47	101	82,79	< 0,0001	chi2
	Yes	7	5,74	3	2,46	4	3,28		
VSGS	No	114	93,44	16	13,11	98	80,33	0,031	Fisher
	Yes	8	6,56	5	4,10	3	2,46		
VCS	No	115	94,26	18	14,75	97	79,51	0,0197	Fisher
	Yes	7	5,74	4	3,28	3	2,46		
Meningitis	No	111	90,98	14	11,47	97	79,51	< 0,0001	Fisher
	Yes	11	9,02	8	6,56	3	2,46		

SD: Standard deviation; WA: Weeks of amenorrhea; EVD: external ventricular shunt; VP shunt: ventriculo peritoneal shunt; VSGS: ventriculo sub galeal shunt; VCS: ventriculocysternostomy

**Table 7:** Risk factors for pejorative motor outcomes on multivariate analysis

Variables		OR	IC	p
Papile grading	3–4 versus 2	2.11	1.2–3.8	0.05
Birth weight	4th quartile versus 2nd quartile	1.37	1.8–3.2	0.44
	3rd quartile versus 2nd quartile	1.41	1.2–2.5	0.46
VCS		0.33	0.04–2.1	0.21
EVD		0.55	0.1–2.8	0.45
VP shunt		0.54	0.8–3.8	0.42
VSGS		0.4	0.05–2.5	0.25
Meningitis		1.17	0.1–1.8	1
Increased head circumference > +2SD		4.15	1.7–10.3	0.007

*VCS*, ventriculocysternostomy; *EVD*, external ventricular shunt; *VP* shunt, ventriculo peritoneal shunt; *VSGS*, ventriculo sub galeal shunt; *SD*, Standard deviation

Among the 77 survivors at 2 years, 37 infants (48.1%) had no association of words at the age of 24 months, 3 (3.9%) suffered from epilepsy, 12 (15.6%) had a visual deficiency and 6 (7.8%) presented hearing impairment.

Among the 22 infants who presented a PHH, 1 died during the study period due to multiple organ failure. Among the 21 survivors, 6, 3 and 1 had a GFCSM score of 2, 3 and 5 respectively. Twelve infants had an association of words at the age of 24 months, 1 suffered from epilepsy, and 2 presented hearing impairment.

## Discussion

In this study, based on the long-term outcomes of 122 newborns with neonatal IVH, we report a PHH rate of 18%. Our result is concordant with data in the literature [2, 17, 18] in which the PHH rate varies between 20 and 35%. Risk factors for PHH were high IVH grade and increased HC at diagnosis.

In a recent study [19] based on the outcomes of 97 infants with neonatal IVH, the PHH rate was of 35%, and the first significant risk factor for PHH was the grade of the initial bleeding. In this series, all infants with a permanent VPS had an initial bleeding grade of III or IV on the Papile classification. In our series, 6 of the 22 infants requiring VP shunt had an initial hemorrhage grade of 2 while most studies limited their inclusion criteria to grade 3 and 4. In another study [2] based on 42 infants with IVH and a PHH rate of 26%, the risk factors for onset of PHH were high IVH grade, late onset (later than 1 week after birth) of bleeding and < 30 WG. The absence of a direct relationship between gestational age at birth and PHH could be due to confounding factors and a higher mortality rate in extreme preterm births. We observed that a HC > + 2 SD at diagnosis was a risk factor for shunt dependence. This observation emphasizes the need for early management of PHH before the onset of ependyma lesions leading to a loss of compliance of the ventricles [13].

The type of CSF derivation device was not a discriminant risk factor for shunt dependence in our cohort. According to current data in the literature, two devices are recommended [12]: the ventriculo subgaleal shunt and the ventricular access device. The use of CSF washing was the subject of an important publication in the year 2003 [20]. The outcomes of this technique were discordant: a higher incidence of secondary bleeding but better neurodevelopmental outcomes at 2-year follow-up [21, 22]. According to a recent meta-analysis [12], there is not a sufficient level of evidence to recommend this strategy. Studies have been conducted to find an alternative to these strategies with the use for example, of iron chelator on animal models [23], to decrease inflammatory response and prevent the onset of hydrocephalus. These strategies could be applied to patients at risk of developing PHH. CSF biomarkers could be of interest to predict the onset of PHH in these young patients. For example, in a recent study, Morales et al. [24], demonstrated a strong association between the CSF level of amyloid precursor protein (APP) and ventricular size.

Concerning mortality, we report a rate of 36.9% defined as the rate of mortality during the 2 years of follow-up. In our study, risk factors for mortality were low gestational age at birth and high IVH grade. This rate is concordant with data in the literature [25]. Death was due to extra neurological causes in more than 50% of cases because of the onset of other complications inherent to prematurity (nosocomial infections, enterocolitis...) of children with a PHH.

Concerning motor outcomes at 2 years, 43 patients had a GMFCS ≥2. Risk factors for negative evolution were high IVH grade on ultrasound and increased cranial circumference at the time of hydrocephalus management. In a serie of 95 patients, De Vries et al. [13] reported motor impairment in 22% of patients with a PHH. In another study [11] based on 6000 patients, of the 40% who reached 2-year survival, 14% presented cerebral palsy. The prognosis was worse in patients with permanent VP shunt. In a previous study with 400 patients [26], the rate of motor impairment was 23%. As in our study, all these retrospective studies observed that the rate of cerebral palsy was elevated if we compared them to the rate of cerebral palsy in the cohorts of preterm infants regardless of the presence or absence of IVH [27]. However it was mentionned in several studies that the higher the grading of IVH, the higher the risk of cerebral palsy. This observation may help to explain the reduced cerebral volume and impaired developmental outcomes in patients with IVH.

In our cohort, 40 infants (51.9%) had an association of words at the age of 24 months. The impact of prematurity and IVH on school performance could not be evaluated in our study. A Dutch series [26] evaluated the neurodevelopmental outcomes of 484 preterm children born before 32 WG. In this cohort, at the age of 2 years, forty-five (15.3%) of the 294 survivors had a minor and 23 (7.8%) a major handicap. The presence of an IVH was associated with impaired neurodevelopmental outcome. The evolution of the same cohort was evaluated at the age of 14 years [28], school performance data were obtained for 278 of the 304 surviving adolescents. In this study, 129 adolescents (46.4%) performed normally, 107 (38.5%) were slow learners and 42 (15.1%) needed special education services. The presence of a perinatal IVH was the only factor, which was significantly asssociated with the need for special education. There was a fourfold risk of special education comparing patients with grade III/IV and patients without IVH. We report a sensorial deficit in 18 infants (23%) in our cohort. The presence of sensorial deficit is of interest and must be diagnosed early because it contributes to poor school performance.

Our study has some limits as it is a retrospective study collecting a high number of preterm infants born during a long period of 14 years during which the standards of care of preterm infants have changed. In our study, there was no difference in the rate of antenatal administration of corticosteroid or magnesium sulfate between groups of children with IVH with or without PHH. Both molecules have been associated with a lower rate of IVH. We can only observe that the rate of antenatal corticosteroid administration was low (52.7%) as well as the rate of antenatal magnesium sulfate (13.1%).

## Conclusion

We conducted a study on 122 patients with a neonatal IVH. Among the 77 surviving patients at 2 years, 22 (18%) required a permanent VP shunt. Clinical evolution was favorable in 38 of the 77 survivors (49.4%). The risk factors for shunt dependence and impaired neurodevelopment were IVH grade and increased head circumference. We emphasize the need for close follow-up of these infants and early surgery in case of hydrocephalus. Among surviving patients, close attention must be given to neurodevelopment because of the risk of long-term consequences associated with this pathology. The development of biomarkers and medical therapeutic strategies may help to predict PHH and reduce its consequences.

## Additional file

Additional file 1: Description of data: clinical and radiological data collected for the study in the 122 newborns patients. (XLSX 48 kb)

## Abbreviations

aOR: Adjusted odds ratio
APP: Amyloid precursor protein
CI: Confidence interval
CSF: Cerebrospinal fluid
cUS: Cranial ultrasound
EGA: Estimated gestational age
EVD: External ventricular drainage
GMFCS: Gross motor function classification system
HC: Head circumference
IVH: Intraventricular hemorrhage
SD: Standard Derivation
VPS: Ventriculoperitoneal shunt
VSGS: Ventriculo subgaleal shunt
WG: Weeks of gestation
